# Animal welfare and zoonosis risk: anti-*Trichinella* antibodies in breeding pigs farmed under controlled housing conditions

**DOI:** 10.1186/s13071-021-04920-1

**Published:** 2021-08-21

**Authors:** Edoardo Pozio, Mario Celli, Alessandra Ludovisi, Maria Interisano, Marco Amati, Maria Angeles Gómez-Morales

**Affiliations:** 1 Department of Infectious Diseases, Istituto Superiore di Sanità, Viale Regina Elena 299, 00161 Rome, Italy; 2 Autonomous Veterinary Practitioner, Forlì, Italy

**Keywords:** *Trichinella*, Serology, Breeding pig, Housing condition, Outdoor access, Animal welfare

## Abstract

**Background:**

Domesticated pigs are the main source of *Trichinella* sp. infections for humans, particularly when reared in backyards or free-ranging. In temperate areas of southern Europe, most pigs are farmed under controlled housing conditions, but sows and sometimes fattening pigs have access to outdoors to improve animal welfare. The aim of the present study was to investigate whether outdoor access of breeding pigs farmed under controlled housing conditions can represent a risk for *Trichinella* sp. transmission when the farm is located in an agricultural area interspersed with wooded areas and badlands, where *Trichinella* spp. could be present in wildlife.

**Methods:**

Serum samples were collected from 63 breeding sows and one boar before and after their access to an open fenced area for 2 months and from 84 pigs that never had outdoor access. Samples were screened for anti-*Trichinella* antibodies by ELISA, and positive sera were confirmed using Western blot (Wb) excretory/secretory antigens. To detect *Trichinella* sp. larvae, muscle tissues from serologically positive and negative pigs were tested by artificial digestion.

**Results:**

Thirteen (20.6%) sows and one boar tested positive with both ELISA and Wb. No larvae were detected in muscle samples of serologically positive and serologically negative pigs. Positive serum samples were then tested by Wb using crude worm extract as antigens. The Wb banding pattern displayed was that characteristic of encapsulated species (*Trichinella spiralis* or *Trichinella britovi*).

**Conclusions:**

The detection of anti-*Trichinella* antibodies without larvae in the pig muscles, supported by epidemiological data, suggests that pigs may have been exposed to *T. britovi*. This study stresses the importance of instigating monitoring systems at farm level to prevent *Trichinella* sp. transmission and to investigate, through a landscape parasitological study, the suitability of a site before the planting of a high containment level pig farm in which the sows can have outside access to improve their welfare during pregnancy.

**Graphical abstract:**

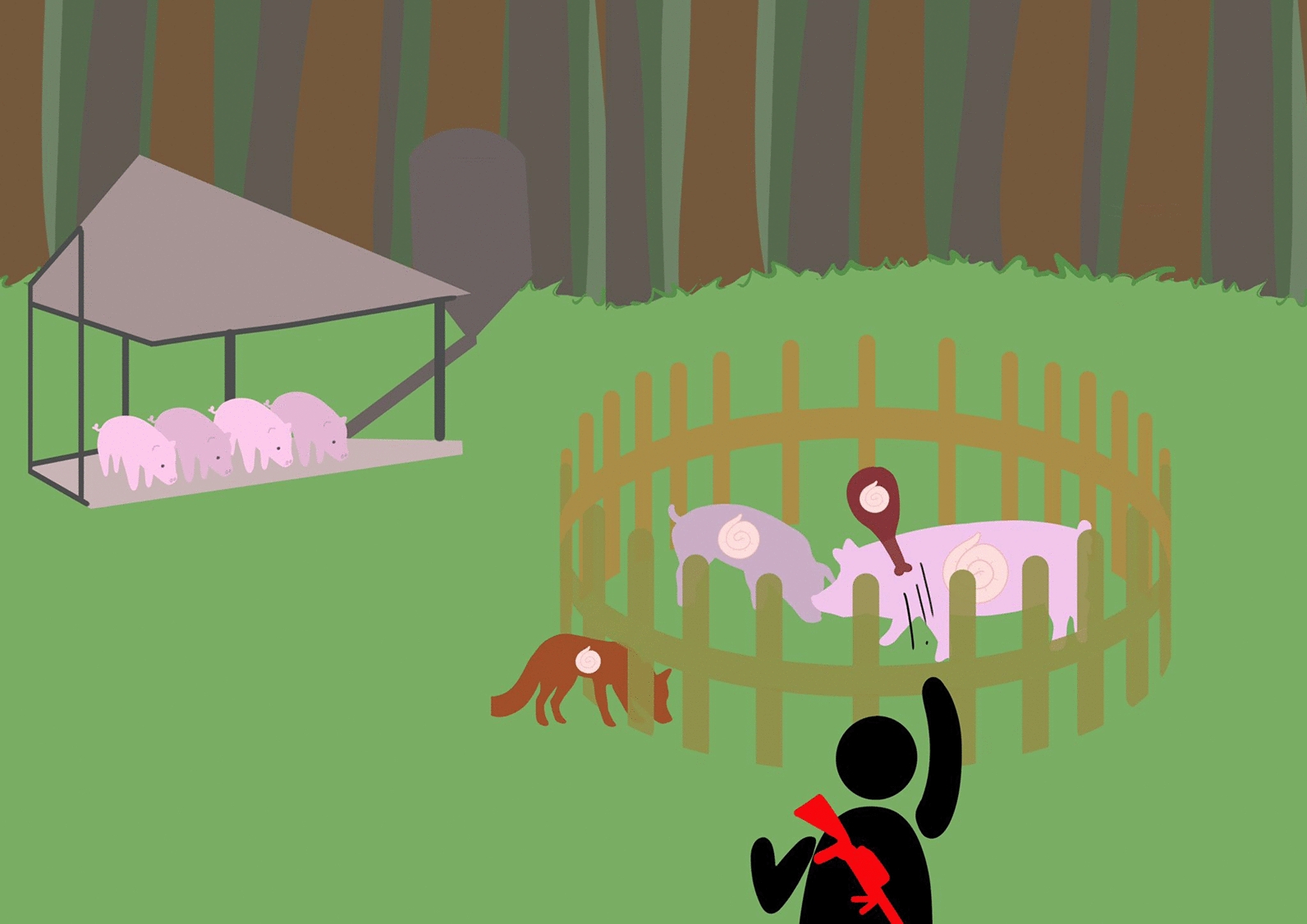

## Background

The history of the zoonotic nematodes of the genus *Trichinella* has been intertwined with that of the domestic pig since 1846 when *Trichinella* larvae were detected in the extensor thigh muscles of a hog [1]. From that moment onwards, there has been a crescendo of epidemiological information, and the pig has been considered the main reservoir host for over a century with pork scraps being identified as the main source in the transmission of these parasites to pigs [2–4]. At the same time, raw or undercooked meat, and meat products from pigs and wild boar, especially if they came from poaching, were the main source of infection for humans [4]. Of the 13 *Trichinella* taxa recognized today, only *Trichinella spiralis* is well adapted to swine in which larvae can survive in its muscles for at least 2 years. Comparatively, the infectivity and larval survival in muscles of other species, e.g. *Trichinella britovi* and *Trichinella pseudospiralis*, are very low [5–7].

In the last 40–50 years, livestock breeding has undergone a remarkable change mainly to increase production and to improve animal welfare. To apply controlled housing conditions, most farmed pigs should not have any outside access; however, in temperate regions (e.g. Mediterranean countries), sows have access to open fenced areas during gestation as well as in holdings applying controlled housing conditions. Based on national histories of organic pig production, diverse climatic conditions and national organic farming regulations, different housing systems are used for keeping pregnant and lactating sows in organic farms in European countries. In some countries, sows are at pasture throughout all stages of pregnancy and lactation. In other countries, most lactating sows are housed indoors. Mixed indoor and outdoor housing systems also exist. Consumers and farmers expect organic farming to ensure high standards of animal health and welfare. Consumers expect pigs to be kept in natural surroundings, such as provided by outdoor systems.

The aim of the present study was to monitor whether sows of a holding applying controlled housing conditions according to the European Commission [8], which had access to an open fenced area during pregnancy, could be at risk of acquiring *Trichinella* infection.

## Methods

### Geographical area and pig farm

**Fig. 1:**
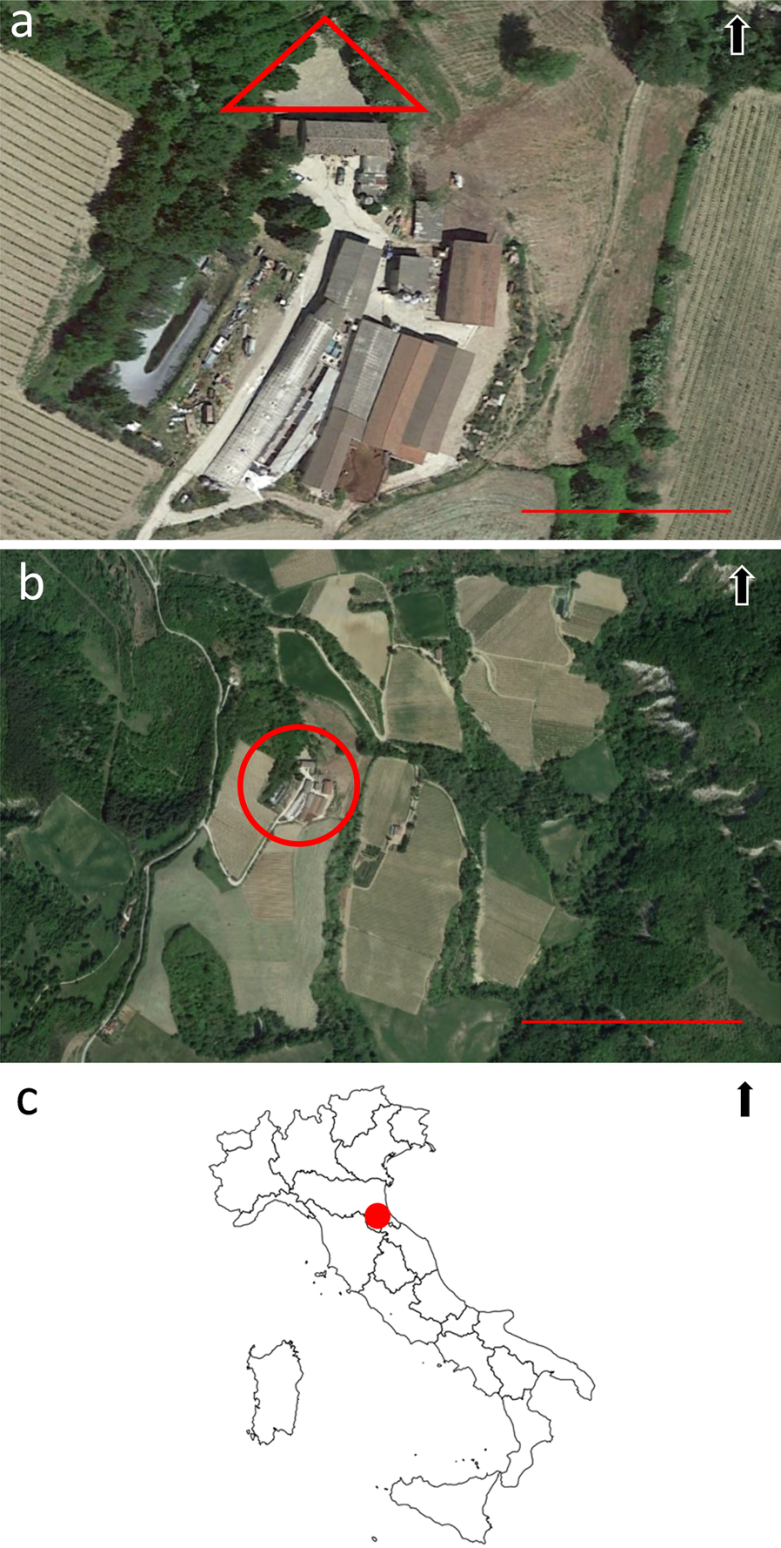
Map of the pig farm and surrounding area (Dovadola municipality, Forlì province), 44.114325°, 11.906907° (downloaded from Google Earth Pro); the black arrows show the north. **a** The investigated pig farm; the red triangle shows the outside fenced area, where sows had access from 40 up to 100 days after fertilization with the boar for the control of estrus in sows; scale bare 50 m. **b** The investigated pig farm (in the red circle) with the surrounding environment showing a wooded area on the left, cultivated fields in the central picture and badlands with minimal vegetation on the right; scale bar 250 m. **c** Map of Italy showing Forlì Province, Emilia Romagna region, Northern Italy

The study was carried out on a farm located on the slopes of Apennines at 360 m above sea level (asl) and surrounded by cultivated fields, small woods of Austrian oak (*Quercus cerris*), common hornbeam (*Carpino betulus*), common hazel (*Corylus avellana*) and badlands with minimal vegetation (Fig. 1). No other farms were present nearby. The nearest village (Dovadola, Forlì province, Emilia Romagna region, Northern Italy) is lineally 1.5 km away. The farm has an average presence of 800 heads and 80 breeding sows and produces fattening pigs and boars. There are masonry shelters for the gestation period with a walking area on fenced land (1.5 m high electrified fence of 10 × 10 cm mesh and 40 cm underground) of about 600 m² (Fig. 1), a shed with delivery, weaning and growth separated areas, insulated fiberglass huts and relatively confined parquet with plasticized grating for weaning-growth, insulated shelters with confined parquets and grated concrete floors for the growth-fattening phase and straw shelters for gilts and sows. In 2017, the competent authority was evaluating whether this farm could be considered officially recognized as a holding applying controlled housing conditions with regard to feeding and housing according to Commission Regulation No. 1375/2015 [8].

### Sample collection

**Fig. 2:**
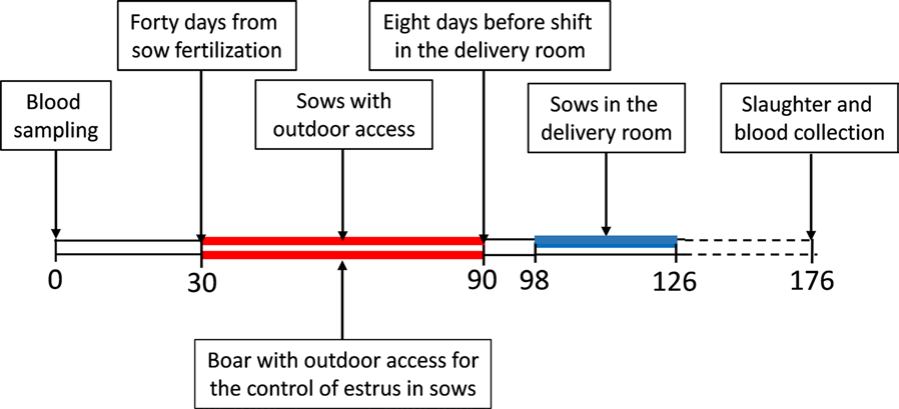
Timeline scheme of sows and boar outdoor access and blood sampling. Red bars: period during which the sows and the boar had access to the walking area: blue bar: sows in the delivery room

In 2017, as part of health checks, blood samples were collected from the jugular vein from breeding sows (*n* = 63, Large White × Duroc) and one boar (Duroc) 30 days before and 86 days after the outdoor access and tested for anti-*Trichinella* antibodies. As shown in Fig. 2, 40 days after fertilization, sows had outdoor access in a fenced area with a boar (for the control of estrus in sows) for a 2-month period. Then, sows were again kept indoors for 8 days before moving to the farrowing room for 4 weeks. As control group, 70 fattening and 14 breeding animals without outdoor access in the study period were also tested. Sera were obtained after centrifugation of clotted blood and kept at − 20 °C until further analysis.

### Serology

**Fig. 3:**
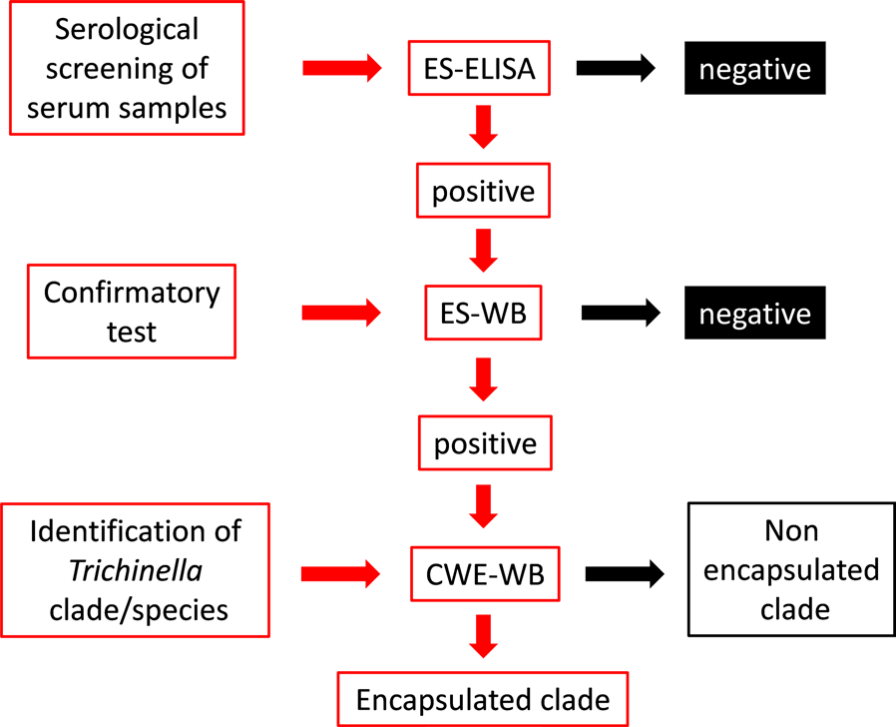
Scheme of serological tests performed to detect anti-*Trichinella* antibodies and to identify the etiological agent at the clade/species level

Two serological tests, enzyme-linked immunosorbent assay (ELISA) and Western blot (Wb) with excretory/secretory (ES) antigens from *T. spiralis* muscle larvae, were used as screening and confirmatory tests, respectively. Wb with a crude worm extract (CWE) from *T. spiralis* muscle larvae was used to identify the etiological agent at the clade/species level, according to the scheme shown in Fig. 3.

### Antigen preparation

The ES antigens were prepared according to a previous published protocol [9]. CWE was prepared from mouse muscle larvae (ML) collected by HCl-pepsin digestion. Following digestion, ML were washed several times using 0.1 M phosphate-buffered saline (PBS), pH 7.2, and stored at − 70 °C in the presence of protease inhibitors (Sigma-Aldrich, Saint Louis, MO, USA). After four thawing/freezing cycles, ML were crushed in a glass potter homogenizer using a Teflon pestle and further disintegrated by sonication. The larval suspension was maintained overnight at 4 °C with magnetic stirring and centrifuged for 1 h at 13,000×*g* at 4 °C. The protein concentration of the supernatant was determined by the Bradford method.

### ELISA

Anti-*Trichinella* IgG of each individual serum sample was tested using a validated ES-ELISA, according to a previously published protocol [9]. Briefly, pig sera were diluted 1:50. Peroxidase-labeled anti-swine IgG was diluted 1:30,000 (Kierkegaard and Perry Laboratories, Gaithersburg, MD, USA). The optical density (OD) was obtained by reading the reaction at 450 nm using an ELISA plate microtiter reader (Dynex Technologies, Chantilly, VA, USA). For each serum sample, an ELISA index (IE) expressed as percentage of positivity was calculated according to Gómez-Morales et al. [9]. The cutoff value, calculated as the mean (+ 3 SD) of the OD values of 880 serum samples from *Trichinella* free pigs, was 18% (data not shown).

### Western blot

ELISA-positive serum samples were tested by a validated ES Wb to confirm positivity [10] and by CWE Wb to identify the etiological agent at the clade/species level [11]. Briefly, 150 μg of total proteins corresponding to ES or CWE antigens was diluted and loaded in 10% pre-cast NuPage Novex Bis-Tris Gels® (Life Technologies, Carlsbad, CA, USA) as reported in the instructions for electrophoresis using the XCellSureLock® Mini-Cell (Life Technologies). Proteins were electrophoretically separated under reducing conditions and transferred to nitrocellulose (Bio-Rad, Hercules, CA, USA) at room temperature (RT) for 1 h. The nitrocellulose filters were blocked with 5% skimmed milk in 1× Tris Borate Saline Tween (TBST, 50 mMTris pH 8.0, 150 m NaCl, 1% Tween 20) at 4 °C overnight and washed three times with 1× TBST. Nitrocellulose filters were cut into strips, each of which was then incubated with swine sera with 3% w/v skimmed milk (Sigma-Aldrich) in 1× TBST at RT for 1 h. After washing three times with 1× TBST, the strips were incubated for 1 h with a 1/3000 dilution of goat anti-pig IgG conjugated with horseradish peroxidase (Biorad). To reveal proteins with high efficiency, the LiteAblot® Plus chemiluminescence system (Euroclone, Pero, Milan, Italy) was added to the strips for 5 min. The proteins were then visualized on a ChemiDoc™ XRS System (Bio-Rad), and images were analyzed using the Image Lab™ software version 4.0 (Bio-Rad).

### Reference sera

Nine serum samples from pigs experimentally infected with *T. spiralis*, *T. britovi* and *T. pseudospiralis*, the three species present in Central and Southern Europe, were used as positive control sera. Twenty serum samples from pigs confirmed as *Trichinella* negative were used as negative controls.

### Artificial digestion

The serologically positive pigs were slaughtered after the end of lactation. Muscle samples were collected from the diaphragm pillars, tongue and masseters and tested by artificial digestion according to a published protocol [8]. Different amounts of muscles were collected from serologically positive (300 g) and serologically negative breeders (10 g) and fattening pigs (1 g).

### Statistical analysis

The sample size of control pigs was calculated with 95% CI, assuming an expected prevalence of 0.01% and a precision of 0.05% [12].

## Results and discussion

**Fig. 4:**
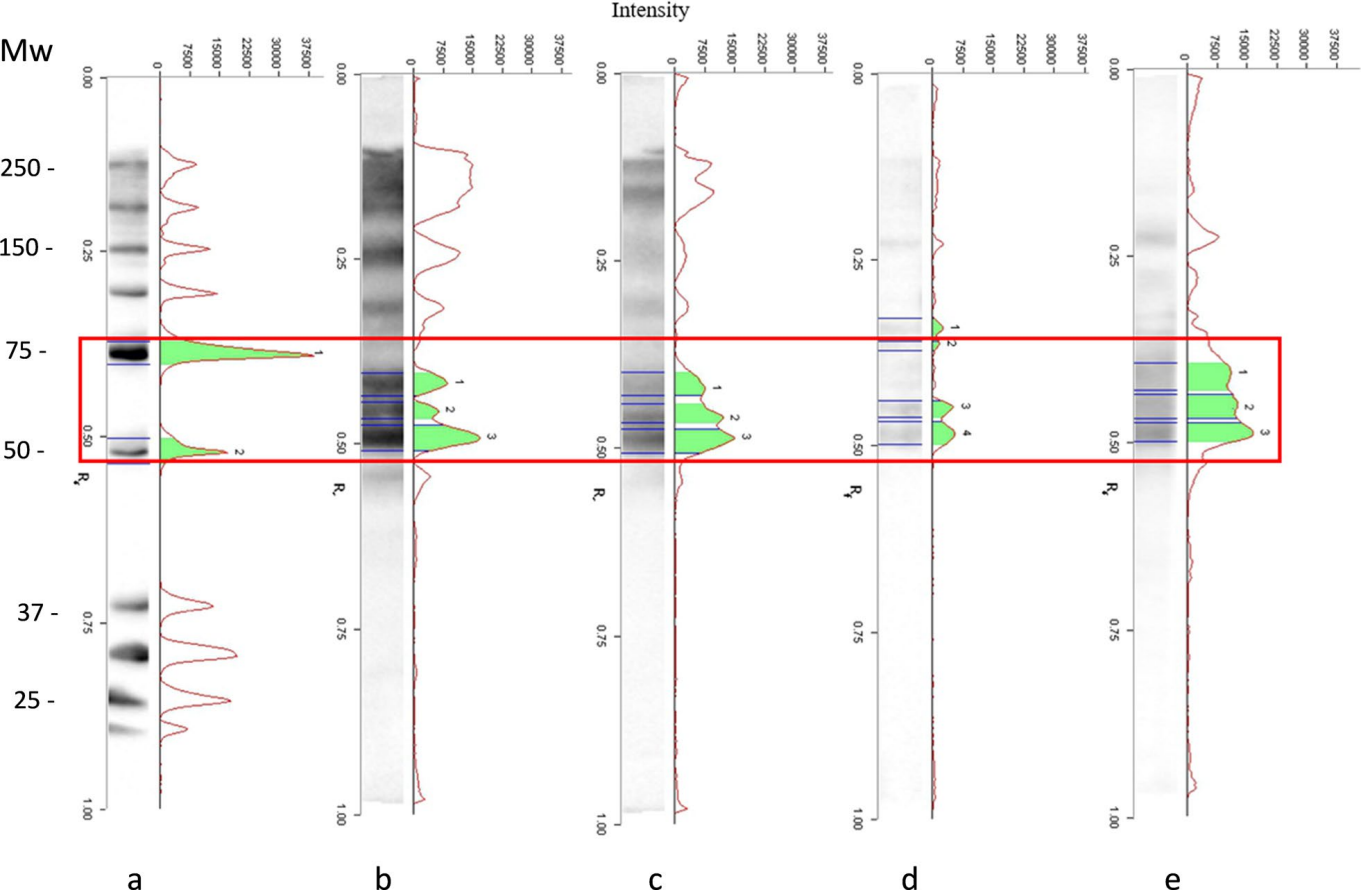
Western blot (Wb) banding patterns of *Trichinella spiralis* crude worm extract (CWE) with sera from serologically *Trichinella*-positive sows. Lane Mw: molecular weights in kDa. **a** Wb pattern of molecular weight markers. **b** Wb pattern of serum from *T. spiralis* experimentally infected pig. **c** Wb pattern of serum from *Trichinella britovi* experimentally infected pig. **d** Wb pattern of serum from *Trichinella pseudospiralis* experimentally infected pig. **e** Wb pattern of serum from one representative sow of the investigated farm. The red box indicates the characteristic diagnostic pattern of recognition for each *Trichinella* species

As shown in Table 1, serum samples collected from 63 sows and 1 boar before their outdoor access tested negative by ELISA, whereas serum samples from 18 sows and 1 boar (29.7%) collected 86 days after their last outdoor access were positive by ELISA. ELISA-positive sera were tested by Wb and 13 breeding sows (20.6% of sows with external access for a period of 2 months; 16.2% of breeding sows present on the farm) and 1 boar were confirmed as positive for anti-*Trichinella* antibodies (Table 1). All serum samples from control pigs, i.e. those without outdoor access in the study period, tested negative by ELISA. The Wb pattern with CWE antigens of serum samples from the 14 Wb-positive sera was consistent with the banding pattern of encapsulated species (*T. spiralis* and *T. britovi*), which is different from that of the non-encapsulated species (e.g. *T. pseudospiralis*) (Fig. 4). No larvae of *Trichinella* sp. were detected by artificial digestion in muscle samples of serologically positive sows and boar and of serologically negative breeding and fattening pigs.

**Table 1:** Serum samples from pigs tested for anti-*Trichinella* antibodies by ELISA and Western blot (Wb) using excretory/secretory antigens (ES) or crude worm extract antigens (CWE)

Pigs	Positive/tested sera by ES-ELISA	Positive/tested sera by ES-Wb	CWE-Wb pattern
Breeding pigs 30 days before outdoor access	0/64	nd^c^	nd
Breeding pigs 86 days after outdoor access	19/64^a^ (29.7%)	14/19^b^ (73.7%)	*T. spiralis* or *T. britovi*
Breeding pigs without outdoor access	0/14	nd	nd
Fattening pigs without outdoor access	0/70	nd	nd

*nd* not done

^a^18/63 breeding sows and 1/1 breeding boar

^b^13/18 breeding sows and 1/1 breeding boar

This study shows the successful application of serology for the surveillance of *Trichinella* sp. infection in pigs with outdoor access. The seroconversion for anti-*Trichinella* IgG in 21.8% of breeding pigs, which had had outdoor access for 2 months, suggests that these animals were exposed to *Trichinella* spp. in the walking fenced area. This assertion is supported by the seronegativity of the control pigs without outdoor access for the entire study period and by the seroconversion of the boar for the control of estrus in sows, i.e. the only animal that had access to the walking area with the sows. The previous year (2016), the sows that had access to the walking area had been monitored as in 2017, but with negative results (data not shown).

Furthermore, pigs were fed exclusively with certified feed, no animals were slaughtered within the farm and breeding pigs at the end of their career, and the fattening pigs were sent for slaughter in certified slaughterhouses for the search for *Trichinella* sp. larvae according to the European legislation [8]. Therefore, at the farm level, there was no risk that the infection could spread among pigs.

The tail-biting, which Zimmermann et al. [13] had hypothesized as a possible route of *T. spiralis* transmission from infected to uninfected pigs on the farm, was never observed among pigs of this farm. Furthermore, the very low larval load per gram of muscle tissue of *T. britovi* compared to that of *T. spiralis* [5–7] makes this route of transmission even more unlikely.

The method of *Trichinella* sp. transmission from wildlife to the sows and boar with outdoor access to the fenced area is unknown. We hypothesize that a *Trichinella* sp. infected carnivore mammal may have passed through the fence (e.g. stone marten, pine marten, weasel) or jumped over the pen (e.g. red fox) and was then killed and devoured by the sows and the boar present in the fenced area. Alternatively, a poacher may have thrown leftovers from the slaughter of a *Trichinella* sp. infected animal (wild boar or carnivore) over the fence. The fenced area is only 140 m far from two inter-estate roads used by hunters. The farm is located on the slopes of Apennines, a mountain area full of wild animals in which *T. britovi* and very seldom *T. pseudospiralis* have been detected [14–18].

The wild boar greatly expanded their distribution areas because of the progressive adaptation to the most varied ecological and environmental conditions linked in most part to modified biological factors. In the last 30 years, the home range of this omnivorous animal has quintupled, involving different geographical areas. In Italy, wild boars are now diffused from lowlands to hilly and mountainous areas; the presence of wild boar has been observed also in the periphery of urban areas [19]. In the same years, the wolf (*Canis lupus*), one of the main reservoir hosts of *T. britovi* in Italy [14], increased and expanded its population in most of the country including the Foreste Casentinesi National Park [20], which is lineally only 20 km away from the farm. The environment between the National Park and the pig farm is covered with woods interspersed with cultivated fields and therefore easy to transit for wild animals.

From 2010 to 2013, *T. pseudospiralis* was detected in four wild boars reared within a fenced area surrounded by a country road in northeastern Italy. The origin of infection of these wild boars was unknown, and the authors argued that a carnivore mammal or bird had entered the fenced area or that hunters or poachers had thrown *T. pseudospiralis* infected scarps or offal of a hunted animal in the fenced area [15, 21].

In Italy, the most widespread *Trichinella* species is *T. britovi* detected in > 97% of approximately 400 isolates of *Trichinella* spp. from wild and domesticated animals, whereas *T. pseudospiralis* and *T. spiralis* were detected in only 2.1% and 0.5% of isolates identified at species level, respectively [18].

The detection of anti-*Trichinella* antibodies with the Wb profile of encapsulated species, in the absence of larvae in the muscles of pigs, which had had access to a walking fenced area from 86 to 146 days earlier, suggests that the sows and the boar could have been exposed to *T. britovi* since: (i) it is the most prevalent species in Italy, as above reported, and in some countries of southern and central Europe (e.g. Portugal, France, Belgium, The Netherlands, Switzerland, Austria, Slovenia, Greece) [22]; (ii) the larvae of this species are known to survive only for a few months in muscles of pigs belonging to the same race (Large White × Duroc) of those of this study and (iii) anti-*Trichinella* antibodies are detectable for at least 2 years after the infection [7]. We believe that *Trichinella* sp. exposure could not have been caused by the ingestion of infected rats as the larvae of *T. britovi* are unable to survive in rat muscles because they quickly undergo a calcification process [23, 24].

Even in regions where the zoonotic risk due to the pork consumption is low, more muscle samples must be tested to detect *Trichinella* sp. infections in breeding pigs than in fattening pigs according to the EU legislation [8]. The access of pigs to an open area represents a high risk for the transmission of zoonotic agents including those of parasitic origin such as *Trichinella* spp. and *Toxoplasma gondii*.

Controlled housing conditions imply that swine housing must include physical barriers that prevent swine from being exposed to wildlife (including birds). Stepwise exclusion is accomplished by creating barriers external to the buildings, such as open spaces which contain no animal harborage and keeping these areas free of vegetation. The creation of space free of any type of vegetation, including grass bounded by a second fence at 5–6 m away from the first fence, is needed to prevent wild and/or synanthropic animals from entry into the farm and to prevent scarps and offal from hunted animals being thrown over the net. Often, gravel is used to line the perimeter of buildings; this enhances other efforts to control rodents [25].

In the present study, sows, even if infected, would not pose a zoonotic risk, since they would have been tested for *Trichinella* spp. at the end of their role as breeding sows, according to the European Commission [8]. Furthermore, if *T. britovi*, as suspected, was the etiological agent, the time lapse between outside access and slaughtering, which included farrowing, suckling and weaning, would have been sufficient for the devitalization of larvae present in their muscles as already observed [7].

The risk for humans to acquire trichinellosis by pork consumption is quite low in Italy. Indeed, *T. spiralis* has been rarely detected in susceptible wild animals (only two reports in red foxes [18]) and never in locally raised livestock. In the past 20 years, only 26 cases of trichinellosis were documented because of the consumption of *T. britovi*-infected pork and pork-related products from pigs reared in the wild in the island of Sardinia compared to about 140 cases of trichinellosis acquired through the consumption of *T. britovi*- or *T. pseudospiralis*-infected wild boar meat from hunting activities during the same period [26, 27] (Gómez-Morales M.A. unpublished data).

Combining three serological tests greatly increases the chance to indirectly acquire epidemiological information on the circulation of *Trichinella* spp. in swine even for species whose larvae have a short survival in the host muscles. To improve animal welfare, food safety and productivity, there is a need to put all suitable measures in place to reduce transmission of zoonotic pathogens. In areas with a mild climate like the Mediterranean countries, the possibility for pigs and sows, in particular, to have access to the outside in fenced areas is very frequent and, unfortunately, sometimes can cause problems regarding animal safety such as the case presented in this study.

## Conclusions

This investigation demonstrates that planning a pig farm under controlled conditions should include an accurate parasitological study of the surrounding habitat in which the farm will be located to reduce the risk of transmission of zoonotic agents. However, while outdoor access poses a risk of infection with *Trichinella* spp., the examination relevant for food safety (i.e. digestion at slaughter) will still ensure consumer safety as required by the European legislation.

## Data Availability

The data supporting the conclusions of this article are included within the article. Raw data presented are available at the Istituto Superiore di Sanità (Dr. Maria Angeles Gomez Morales, email: mariaangeles.gomezmorales@iss.it).

## Abbreviations

ELISA: Enzyme-linked immunosorbent assay
Wb: Western blot
ES: Excretory/secretory antigens
CWE: Crude worm extract
OD: Optical density
IE: ELISA index
ML: Muscle larvae
